# First report of white piedra caused by *Cutaneotrichosporon debeurmannianum*

**DOI:** 10.1590/S1678-9946202466060

**Published:** 2024-10-11

**Authors:** Hiram Larangeira de Almeida, Eduardo Camargo Faria, Thales Moura de Assis, Ingrid Gonçalves Costa Leite, Viviane Mazo Fávero Gimenes

**Affiliations:** 1 Universidade Católica de Pelotas, Programa de Pós-Graduação em Saúde e Comportamento, Pelotas, Rio Grande do Sul, Brazil; 2 Universidade de São Paulo, Faculdade de Medicina, Instituto de Medicina Tropical de São Paulo, Laboratório de Imunologia e Micologia (LIM-53), São Paulo, São Paulo, Brazil; 3 Universidade de São Paulo, Faculdade de Odontologia, Programa de Pós-Graduação em Diagnóstico Oral, Radiologia Odontologica e Imaginologia,São Paulo, São Paulo, Brazil

**Keywords:** Cutaneotrichosporon debeurmannianum, White piedra, Scanning electron microscopy

## Abstract

Piedras are small nodules found on the hair shafts. White piedra was initially described as caused by *Trichosporon beigelii*, which was later reclassified in several species. We describe the first case of white piedra caused by *Cutaneotrichosporon debeurmannianum.* Affected hairs were examined *in natura* with scanning electron microscopy, after gold metallization. The typical whitish cerebriform creamy colony was obtained in Sabouraud medium. Fungal genomic DNA extracted from cultures and locus was amplified with the NL1/NL4 primer pair from the D1/D2 region of the large ribosomal subunit (LSU) of 28S rRNA. With scanning electron microscopy, nodules are easily identified surrounding the hair shaft; with high magnifications, rounded structures adhered to each other were identified. Comparison of the nucleotide sequences of IMT-1703 *Cutaneotrichosporon debeurmannianum* revealed 99.6% similarity with the 28S large ribosomal unit rDNA sequence. This case of white piedra was caused by *Cutaneotrichosporon debeurmannianum.*

## INTRODUCTION

Piedras are a well-known group of trichoses, characterized by the formation of small nodules on the hair shaft. As suggested by its name, the black variant is caused by a melanized fungus, *Piedraia hortae* ^1^, while white variant is caused by several etiological agents^2,3^. Microscopic examination and trichoscopy can be employed to establish the diagnosis^4^. For both variants, ultrastructural studies show fungal structures adhered to the extracellular matrix surrounding the hair shafts^5,6^. White piedra was initially described as caused by *Trichosporon beigelii*, which is a genus characterized by whitish colonies and the formation of blastoconidia and arthrospores, and has been constantly reclassified^2,3^. We examined a 10-year-old girl from a tropical region in Brazil, who presented nodules on the scalp hairs.

## MATERIALS AND METHODS

For causative agent identification, some affected hairs were inoculated in Sabouraud medium. Affected hairs were examined *in natura* with a Jeol scanning electron microscope (JSM - 6610LV), after gold metallization.

For PCR identification and sequencing, the culture was maintained in Sabouraud agar at 30 °C, and genomic DNA was extracted^7^. The fungal locus was amplified with the NL1/NL4 primer pair from the D1/D2 region of the large ribosomal subunit (LSU) of 28S rRNA to a final volume of 50 uL. Each reaction contained 1.25 U of Taq polymerase (Invitrogen, USA); 5 µL of Buffer 10X (Invitrogen); 1.5 mM MgCl2 (Invitrogen); 200 µM dNTPs (GE Healthcare, United Kingdom), and 20 ng of extracted DNA.

Amplification reactions were performed in a Veriti thermocycler (Applied Biosystems, USA) following the conditions of 28S ribosomal RNA, as follows: Initial stage: 95 °C for 5 min; Cycling – 35 cycles of: 95 °C for 45 s; 52 °C for 30 s; 72 °C for 45 s. Final stage: 72 °C for 5 min. The primers used were: NL1 5’ GCATATCAATAAGCGGAGGAAAAG-3’ and NL4 5’ GGTCCGTGTTTCAAGACGG-3’/ Invitrogen, diluted to a concentration of 20 µM. The reaction contained a negative control with the same reagents except for DNA.

The *Trichosporon coremiiforme* strain (IMT-38) was used as a positive control for the reaction. The products amplified with NL1/NL4 were purified using the ExoSAP-IT^TM^ enzyme (Applied Biosystems) in a Veriti thermal cycler (Applied Biosystems, USA). DNA sequencing was performed using the Sanger method; the reactions were performed with the BigDye^TM^ Terminator v3.1 Cycle Sequencing Kit. The sequences obtained were analyzed using the BioEdit (version 7.2.3, Informer Technologies) and subjected to analysis in the GenBank database using the Basic Local Alignment Search Tool (blastn)^8^.

The phylogenetic analysis of the isolate was performed using the maximum likelihood estimation (ML), with the Tamura–Nei evolutionary model, with 1000 bootstrap replications using MEGA version 11 software.

## RESULTS

**Figure 1:**
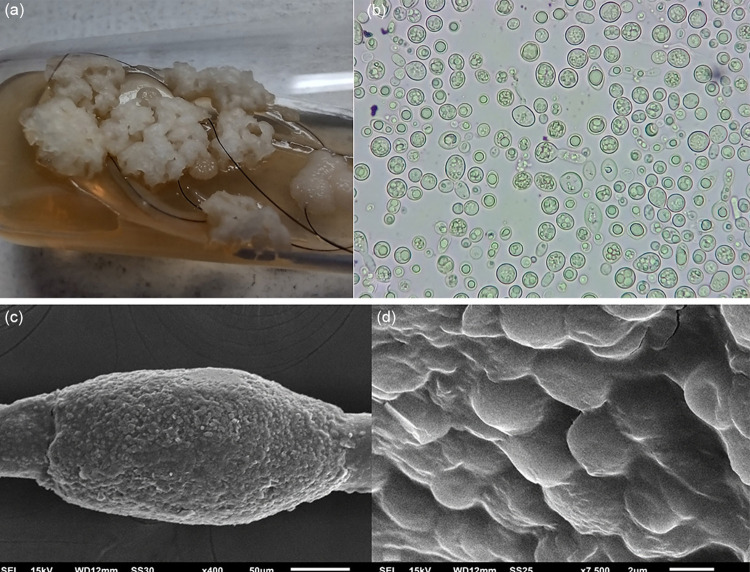
(a) whitish cerebriform creamy fungal colony; (b) microsocopic aspect with predominance of blastoconidia; (c) scanning electron microscopy, low magnification with a nodule surrounding the hair shaft (× 400); (d) scanning electron microscopy, high magnification, detailing the fungal structures (× 7,500).

The typical whitish cerebriform creamy colony^9^ was obtained in Sabouraud medium (Figure 1a), and microscopic examination demonstrated a predominance of rounded structures typical of blastoconidia (Figure 1b).

With scanning electron microscopy with small magnifications, nodules were easily identified surrounding the hair shaft (Figure 1c); with high magnifications, rounded structures adhered to each other were identified on the nodule’s surface (Figure 1d), similar to those observed in optical microscopy of the culture.

**Figure 2:**
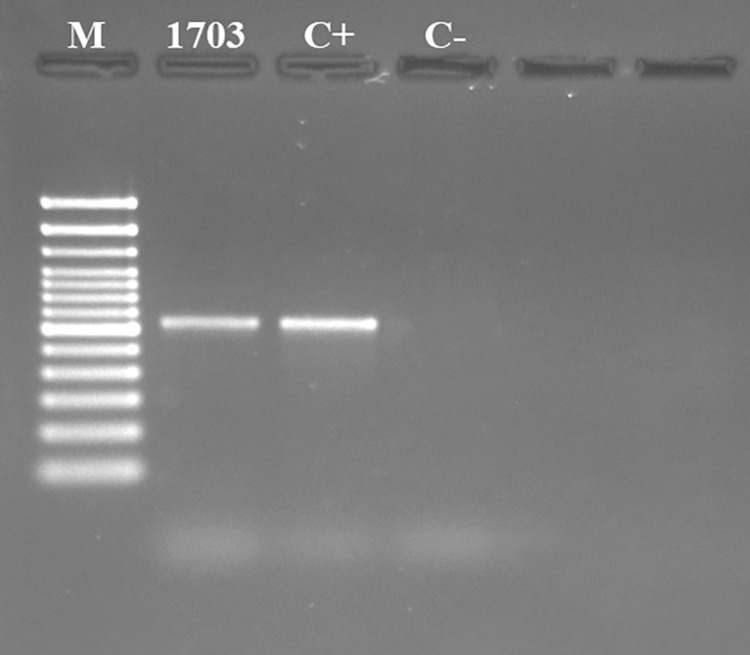
Agarose gel electrophoresis (Invitrogen, USA) at 2% showing the amplification products of isolates IMT1703 (600bp), positive controls (C+, IMT38), and negative control (C-) with primers NL1 and NL4 (Kurtzman, C. P., and C. J. Robnett. 1997). M = Molecular weight marker (Invitrogen, USA).

**Figure 3:**
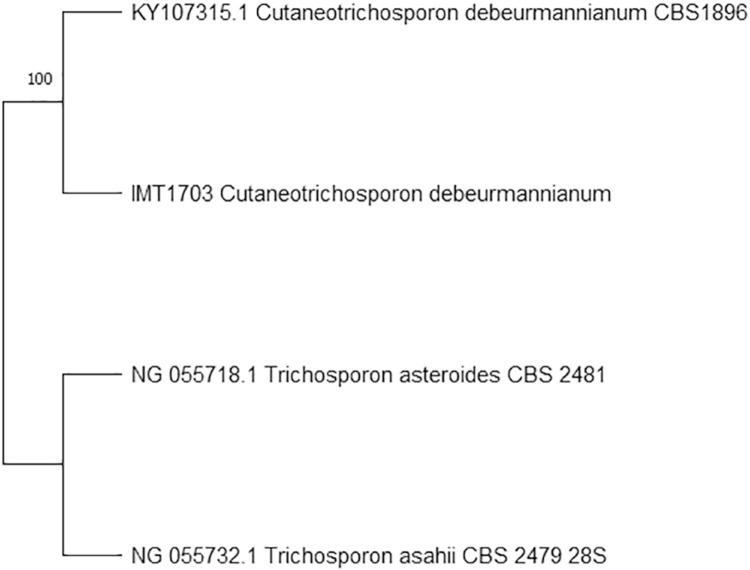
Phylogenetic tree of isolate IMT1703 from the D1/D2 region of LSU, obtained by the maximum likelihood (ML) with 1000 bootstrap replications. The accession numbers of the strains Genbank are indicated on the taxon labels. *Trichosporon asteroides* and *T. asahii* are the outgroup. Sequence analysis obtained after amplification of the DI/D2 region of LSU showed 100% identity with the Genbank reference strain under the access Nº KY107315.1.

Comparison of the nucleotide sequences of IMT-1703 *Cutaneotrichosporon debeurmannianum* revealed 99.6% similarity with the 28S large ribosomal unit rDNA sequence (accession Nº KY107315.1, *Cutaneotrichosporon debeurmannianum* CBS 1896) (Figure 2 and Figure 3).

## DISCUSSION

Several species of *Trichosporon* have already been associated to white piedra, such as*: T. inkin, T. cutaneum, T. mucoides, T. ovoides*, and *T. asahii* ^3^, of which most cases reported *T. inkin* ^10-14^. Interestingly, there are reports of white piedra also caused by fungi from other genus, such as *Cladosporium cladosporioides* ^15^ and *Candida parapsilosis* ^16^. *Cutaneotrichosporon debeurmannianum* (called *Trichosporon debeurmannianum* by some authors) was described in 2001^17^, a study reports a case of skin infection in an immunocompetent patient caused by this agent^18^. The constant changes in the taxonomy of this group^19,20^ hinders reviewing the subject, because several publications still use the former nomenclature.

## CONCLUSION

Our findings describe another causative agent of white piedra.
